# Berlin Heart EXCOR® pediatric ventricular assist device in a patient with Sotos syndrome: a case report

**DOI:** 10.1186/s13256-019-2190-9

**Published:** 2019-08-31

**Authors:** Rita Gravino, Giuseppe Limongelli, Andrea Petraio, Daniele Masarone, Maria Giovanna Russo, Ciro Maiello, Marina Verrengia, Danilo De Paulis, Giuseppe Pacileo

**Affiliations:** 1 Department of Heart Failure Unit, Monaldi Hospital, via Leonardo Bianchi, 80131 Naples, Italy; 2 Department of Pediatric Cardiology Unit, Second University of Naples, Monaldi Hospital, Naples, Italy; 3 Department of Cardiac Transplant Unit, Monaldi Hospital, Naples, Italy; 4 Department of Neurosurgery, San Anna & San Sebastiano City Hospital Caserta, Caserta, Italy

**Keywords:** Sotos syndrome, *NSD1* gene, Berlin Heart EXCOR® pediatric ventricular assist device

## Abstract

**Introduction:**

Berlin Heart EXCOR® pediatric ventricular assist device is a mechanical circulatory support device currently used in pediatric patients. Sotos syndrome is a well-described multiple anomaly syndrome characterized by overgrowth, distinctive craniofacial appearance, cardiac abnormalities, and variable learning disabilities.

**Case presentation:**

We describe a 7-year-old female Caucasian child with classic Sotos syndrome features subjected to implantation of Berlin Heart EXCOR® pediatric biventricular assist device mechanical support. A heart transplant was carried out after a support time of 459 days. After 5 years of follow-up, our patient is clinically stable and the performance of the transplanted heart is excellent.

**Conclusion:**

This case confirms that Berlin Heart EXCOR® pediatric ventricular assist device can provide satisfactory and safe circulatory support for children with end-stage heart diseases, even in those with Sotos syndrome. The syndrome is not a contraindication to implantation, since the complications are the same as those observed in patients without the syndrome and the prognosis is not affected by the disease.

## Introduction

Berlin Heart EXCOR® pediatric ventricular assist device (VAD) is a mechanical circulatory support device currently used in pediatric patients. Sotos syndrome is a well-described multiple anomaly syndrome characterized by overgrowth, distinctive craniofacial appearance, cardiac abnormalities, and variable learning disabilities. The diagnosis of Sotos syndrome relied solely on these clinical criteria until haploinsufficiency of the *NSD1* gene was identified as causative.

We describe a 7-year-old child with classic features of Sotos syndrome (macrocephaly, tall stature, learning disabilities, advanced bone age, and characteristic craniofacial appearance) but without pathogenic *NSD1* mutation (suspected mosaicism), who was subjected to implantation of biventricular support while waiting for heart transplantation.

## Case presentation

**Fig. 1:**
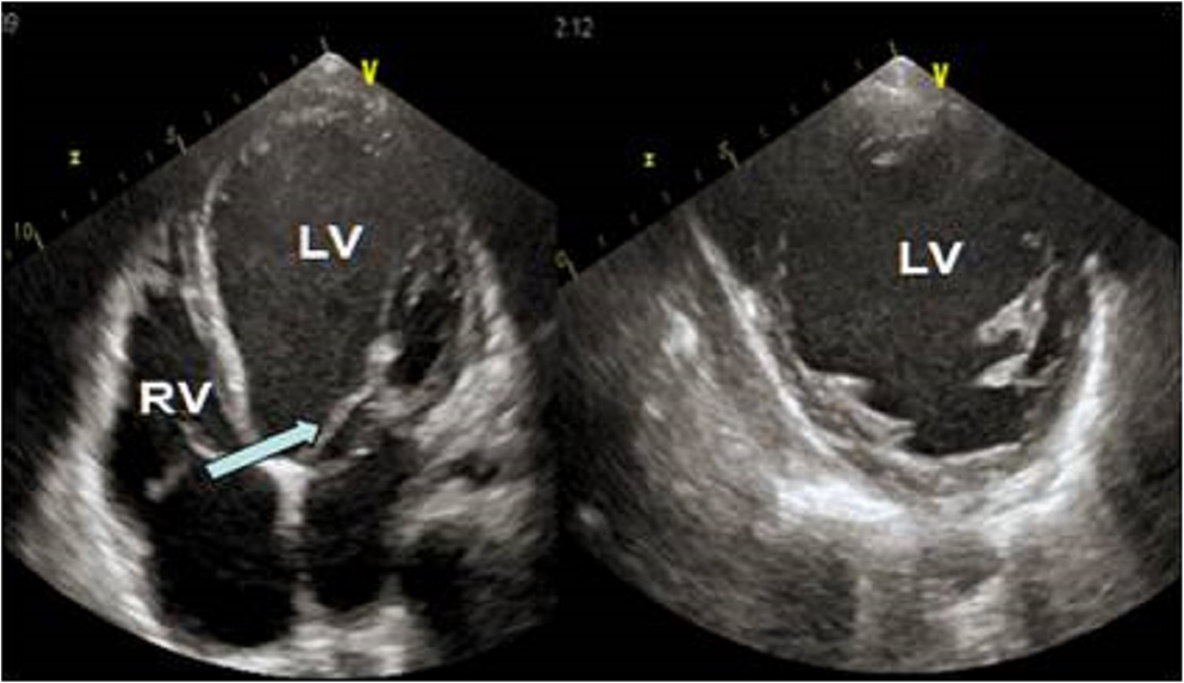
Pre-implantation echocardiographic images. Dilatation of the left ventricle and right ventricle, results of mitral valvuloplasty (*arrow*). *LV* left ventricle, *RV* right ventricle

**Fig. 2:**
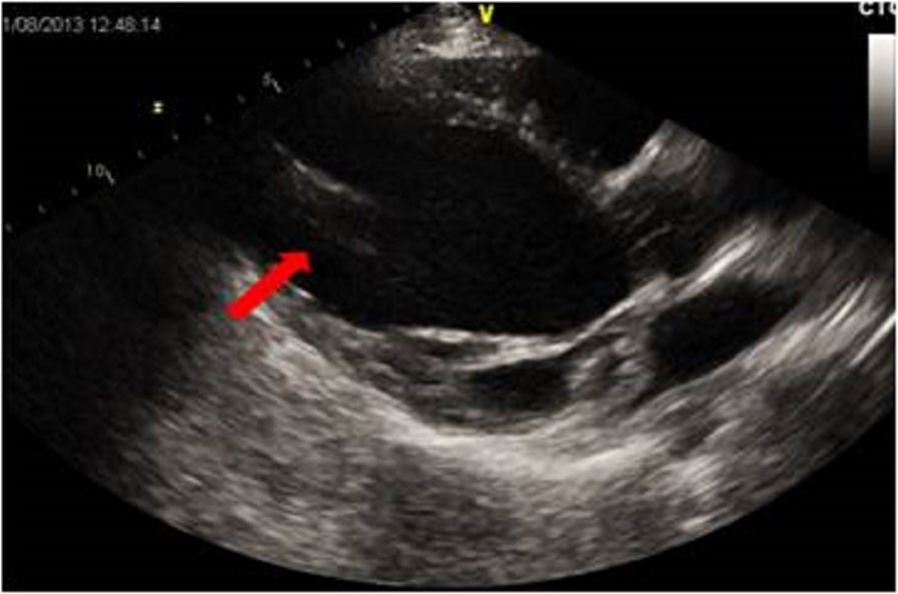
Post-implantation echocardiographic image. Cannula in the left ventricular apex (*arrow*)

At the age of 9 months our female Caucasian patient, who had Sotos syndrome, underwent surgical ventriculoperitoneal shunt for hydrocephalus that was repeated at the age of 5 years. In January 2012, when our patient was 9-years old, an echocardiogram showed left ventricular (LV) dilatation due to floppy mitral valve with moderate-to-severe regurgitation showing normal ejection fraction. As a consequence, she underwent mitral valvuloplasty with excellent immediate results. After 3 months, a significant decrease of global LV pump function was highlighted (Fig. 1). The child was then subjected to drug therapy with inotropic drugs (dobutamine at the beginning, two cycles of enoximone and one cycle of levosimendan) without any clinical improvement. Inflammatory markers were negative and the results of all the tests for infection we performed (nasal and rectal swab, sputum analysis, and urine and blood cultures) were negative too. We did not take into account implantation of extracorporeal membrane oxygenation (ECMO) in order not to compromise the immune status of our patient who was waiting for transplant. After an antibiotic prophylaxis with vancomycin and gentamicin, in September 2012 our patient underwent Berlin Heart EXCOR® pediatric biventricular assist device (bi-VAD) implantation. An endomyocardial biopsy was performed at VAD implantation but only a diffuse fibrotic replacement without signs of active inflammation was in evidence. The size of the pumps was selected on the basis of the body surface area (BSA; right pump, 60 ml; left pump, 80 ml). Implantation was done under transesophageal echocardiogram guidance according to the manufacturer’s instructions: the inflow cannula was implanted at the apex of the left ventricle, and the outflow graft anastomosed to the ascending aorta. On the right side, the inflow cannula was implanted in the right atrium, and the outflow cannula anastomosed to the pulmonary trunk (Fig. 2). The ventriculoperitoneal shunt did not represent any hindrance to the implantation of cannulae. In the immediate postoperative period, epinephrine and nitric oxide were administered and continuous veno-venous hemofiltration (CVVH) was started. Unfractionated heparin was started after 24 hours, and was converted to orally administered vitamin K antagonists and aspirin after 72 hours. ASPItest, thromboelastography, and elastometry were performed to evaluate the coagulation status of our patient but, despite their results, clopidogrel was not added because of nasal, gingival, and wound bleeding with only the dual therapy. After a few days, a wound infection (*Staphylococcus epidermidis*) was found and, as a consequence, antibiotic therapy was established. Despite treatments with orally administered anticoagulants and aspirin, our patient had a cerebrovascular ischemic event with residual hemiplegia after 2 months. Afterward, we tried to insert clopidogrel ​​but, due to a new massive gingival bleeding caused by bone exposure for gingival reduction, it was suspended and a dental reclamation was needed. During the assistance period, sepsis occurred and a change of the pump was necessary three times (twice due to pump chamber thrombosis and once due to infection). Subsequently, recurrent cannula infections developed and were successfully treated with intravenously administered antibiotics and local treatment. The infections recurred until heart transplant which was carried out after a support time of 459 days. After heart transplant, a temporary ECMO was needed for 3 weeks because of graft failure (too small for the body surface) and because of lung trauma that occurred during the intervention. After 5 years of follow-up, our patient is clinically stable and the performance of the implanted heart is excellent.

## Discussion

Systolic heart failure in adults is both highly prevalent and fatal, accounting for 280,000 deaths annually in the USA [1]. Mechanical circulatory support is an effective therapy for adults with advanced heart failure, with 1-year survival rates of 75 to 80% [2]. Heart failure in children is much less common than in adults, but it is highly lethal. Heart transplantation offers effective palliation with 1-year and 3-year survival estimates of 89% and 83%, respectively [3]. Donor availability limits the application of heart transplantation in children and prolongs the waiting period. In infants, the median waiting duration for a donor is 119 days [3]. Overall, the waiting list mortality rate is reported as 12–17%, increasing to 23% for infants [4–6]. Unlike adults, options for mechanical circulatory support as a bridge to transplantation for the pediatric population are limited. The only approved device, DeBakey VAD® *Child* (MicroMed Technology, Inc., Houston, TX, USA), is for larger children (BSA > 0.7 m^2^), and has had limited clinical application [7]. Smaller patients, with the highest waiting list mortality rate, can only be supported by ECMO, a decade-old therapy that offers 40–60% survival to transplant. Its period of support, typically limited to only 10–20 days, is inadequate considering current waiting times and the deterioration of the patient while being supported.

Berlin Heart EXCOR® pediatric was developed in conjunction with Deutsches Herzzentrum (Berlin, Germany) in the early 1990s, as a paracorporeal, pneumatically driven, pulsatile flow mechanical circulatory support device available in sizes virtually suitable for all children. European studies suggested that EXCOR® pediatric can provide stable circulatory support for up to 1041 days in children as old as 6 days and as small as 3 kg [8]. Implantation of a pulsatile, paracorporeal VAD proved to be advantageous in many ways, by improving tissue perfusion and patient mobility, and reversing end-organ dysfunction [9, 10]. VAD Berlin Heart EXCOR® pediatric(s) provides an effective means of bridging children of almost all ages and sizes to cardiac transplantation or myocardial recovery. Sandica *et al.*, reviewing their results regarding the safety of long-term support and outcome, reported a survival rate of 91.6% [11]. The timing of device implantation is crucially important. A team decision is mandatory, but the surgeon has a leading role. A late implantation increases the stress on end-organs and the stress on the right ventricle with an increasing need for bi-VAD support. Time will change a left-VAD (l-VAD) candidate to a bi-VAD candidate, and finally to an ECMO candidate, because the patient may later develop cardiorespiratory failure [11]. In a prospective trial, Fraser *et al.* compared outcomes in patients who had received a VAD with patients in propensity score-matched, historical control groups of children who had received support with ECMO, which is the only other option for mechanical circulatory support currently available for small children [12]. They found that the rate of survival to device explantation (owing to either transplantation or recovery) was markedly higher in the VAD group compared with the ECMO group. In addition, unlike in the ECMO group, in the VAD group an acceptable neurologic outcome was found [12]. Imamura *et al.* demonstrated that in children requiring bridge to transplantation, EXCOR® provided substantially longer support times with a higher survival rate compared to the ECMO group [13]. Finally, Almond *et al*. reported that three-quarters of children survived to transplantation or recovery [14]. In addition, these data support the evidence that today EXCOR® represents a new standard treatment in the USA for pediatric bridge to transplantation.

Mechanical bridge to heart transplantation is also possible in the most difficult category of patients ≤ 10 kg, with outcomes comparable to those of larger children and adults [15]. Berlin Heart EXCOR® pediatric biventricular use in the UK has allowed a significant increase in both the number of children with end-stage heart failure who can be successfully bridged to transplant, and the length of time that they can be supported [16].

However, potential adverse events such as stroke or visceral thromboembolism may be extremely serious. Stiller *et al.* suggested that anticoagulation should be performed with heparin, or Coumadin (warfarin), combined with acetylsalicylic acid (ASA) and dipyridamole, in doses related to daily performed anticoagulation tests [17]. Nevertheless, the risk for cerebrovascular accidents (CVAs) in children with VAD is still high, ranging from 15 to 40% of patients [13, 18–20]. The incidence of CVAs has decreased with institutional experience. Byrnes *et al*. demonstrated in their single-center experience an incremental reduction in CVAs among pediatric patients supported with Berlin EXCOR® VAD showing a current CVA incidence of 16% [21]. In order to decrease the infective complication rate, Stiller *et al.* suggested, as routine procedure, a second-generation cephalosporin for the first week with the addition of vancomycin if the chest remains open; after the first week, antibiotics should be continued in the presence of suspected or proven infection [17]. Further studies are warranted in pediatric patients supported by Berlin EXCOR® VAD to confirm these findings in a larger cohort. Despite the relevant incidence of CVAs, only a small cohort of the assisted pediatric patients suffers from permanent neurologic dysfunction.

The clinical features in children suggestive of Sotos syndrome, that is a childhood overgrowth condition, were first described in 1964 by Sotos *et al*. [22, 23], although the first description of a patient may have been reported in 1931 [24]. The four major diagnostic criteria were established in 1994 by Cole and Hughes [25], based on the systematic assessment of 41 typical cases: overgrowth with advanced bone age, macrocephaly, characteristic facial appearance, and learning difficulties. These clinical criteria remained the cornerstone for the diagnosis of Sotos syndrome until 2002. The recent identification of *NSD1* mutations and deletions [26] has allowed re-evaluation of the features of this condition [27–30]. In 2005, Tatton-Brown *et al*. and the Childhood Overgrowth Collaboration Consortium reviewed the clinical features of 239 cases of Sotos syndrome with *NSD1* abnormalities [31]. They confirmed that overgrowth (including height and occipito-frontal circumference), dysmorphism, and learning disabilities were present in 90% of these *NSD1*-positive individuals, with a wide spectrum of associated features including macrocephaly, advanced bone age, neonatal jaundice and hypotonia, seizures, scoliosis, cardiac defects, and genitourinary anomalies [31]. However, 7 to 35% of the patients with Sotos syndrome of the reported series did not have any *NSD1* anomalies as in the reported case [32].

Our patient had most of the clinical conditions, so the diagnosis was suspected at birth. She had typical facial features characterized by a high anterior hairline, macrocephaly, frontal bossing, a long thin face, frontotemporal hair scarcity, down slanting palpebral fissures, a prominent mandible, and large hands and feet [31–33].

The overall incidence of congenital heart defects reported in some studies of patients with Sotos syndrome is variable (from 8% to 23.5%) [30, 33–36]. The most common defects are septal defects and patent arterial ducts. Structural abnormalities of the mitral valve and mitral regurgitation, as reported in this case report, are rarely described.

Our patient had no abnormalities of the genitourinary system commonly described in Sotos syndrome, which could complicate management and clinical course [37].

In Sotos syndrome, dilatation of the cerebral ventricles is common [38] and hydrocephalus requires treatment with ventriculoperitoneal shunt. The presence of the shunt did not compromise the implantation of the device in our patient. As in most patients with Sotos syndrome, a mild psychomotor impairment was found but it did not represent a contraindication for implantation and for heart transplantation.

In these patients, coagulation abnormalities, such as to predispose to ischemic events and bleeding, have never been reported. In our case, the anatomy of the mandible probably favored the gingival bleeding that occurred.

Like other overgrowth syndromes, Sotos syndrome is associated with a risk of tumorigenesis. The increased risk of tumors was initially calculated at 6–7% [39], but other data suggested 2–3% [40]. The variety of malignancies and their site of origin, as well as the relatively older age of onset compared with other overgrowth syndromes, make screening difficult and the risk is not predictable.

The absence of serious comorbidities, the mild psychomotor impairment, and the unpredictable risk of cancer did not compromise our patient’s possibility of transplantation. Complications were not closely related to the syndrome.

## Conclusions

In agreement with the literature, this case confirms that Berlin Heart EXCOR® pediatric VAD can provide satisfactory and safe circulatory support for children with end-stage heart diseases, even in those with Sotos syndrome. Sotos syndrome is not a contraindication to its implantation; the complications are the same as those observed in patients without Sotos syndrome and the prognosis is not affected by the disease.

## Data Availability

The datasets used and/or analyzed during the current study are available from the corresponding author on reasonable request.
